# 

**Published:** 1854-12

**Authors:** 


### INDEX TO VOL. III. NEW SERIES.

Aphonia of four years cured by electro magnetism, page 22
Alum as an emetic, 16
An Address before the Pennsylvania Society of Dental Surgery, 41
American Medical Association, 141, 518, 280, 46, 136, 225
Alcoholic liquors, Carpenter on, 85
Ammonia, urate of, 88
Academy of Sciences, 89
Arsenic, treatment of poisoning by, 100
Address, valedictory, by Prof. J. V. Z. Blaney, 113
American Medical Monthly, 135
Anatomy, a text book of, 180
Acute ophthalmitis, by S. W. Thompson, M.D., 361
Address, by J. W. Spaulding, M.D., 404
Amputation primary and secondary 409
Auscultation and percussion, 325, 459
Animal and vegetable physiology, principles of, 467
Abscess, hepatic, by F. R. Payne, M.D. 495
Animal heat, a new theory of, 505
Apoplexy, 511
An Address, by John S. Dewey, 541
Anæsthesia, local, 561
Blood, Bernard and Robin on, 561
Brain, injury of, with loss of substance, by B. Woodward, 499
Belladonna, external application of in delirium tremens, 501
Bennett on the uterus, 86

Cholera epidemic, 310, 302, 274, 286, 365, 370, 502
Consciousness in Convulsions and Anæsthesia, 13
Cholera, external stimulants in, 21
Chicago, sanitary characteristics of in 1853, 65
Chemistry for students, Fowne's, 86
Chronic pleurisy. clinical report on, by A Flint, 128
Caries of both hip-joints. 149
Cook County Medical Society. 188
Collodion in erysipelas, by H. Nance, M.D., 196
Case from my Note-Book, by G. H. Hinsey, M.D. 201
Chloroform in obstetrics, 216
Chloride o sodium in intermittent fever, 217. 456
Chemistry, Hand-Book of. 368
Cod-liver oil, effects of on the blood. 413
Cancer and tubercle, concurrent development of, 414
Clinical instruction, 570, 477
Creosote in ophthalmia, by E. R. Rich, M.D 443
Comparative physiology, principles of, 461
Constipation, 507

Diseases of children, a practical treatise on, 47
Diseases of the skin, manual of. 81
Diarrhoea, efficacy of sulphuric acid in, 160, 216
Diseases of the liver, 368
Diaphragmatic hernia, a treatise on, 565

Epidemics and sanitary condition of Chicago, 481, 251
Æsculapian Medical Society, 567, 283
Editorial gleanings, 469
Epidemeological Society, 411
Editorial change, 288. 141
Experimental Researches, by Daniel Brainard, M.D. 352
Erysipelas, the treatment of analyzed, 171
Ellis' Medical Formulary, 84
Emphysema, traumatic, 17
Elimination of lead by iodide of potassium, 20
Experimental Researches applied to physiology and pathology, 36

Fibro-bronchitis and rheumatic pneumonia, 85

Gangrene of the lungs treated by terebinthinate inhalations. 510
General pathology, a treatise on, 124
Gun-shot wound, by S. O. Long, M.D. 2

Hydrophobia, case of, by T. D. Washburn, M.D. 318
Hydrocyanic acid, recovery after large doses of, 157
Hand-Book of Chemistry, 368
Hysteria, 261
Hydatids uterine, by D. W. Young, M.D. 401
Hip disease, by R. L. Hale, M.D. 433
Humbug, Medical, 476
Hospitals for the insane, their organization and arrangement, 519

Indiana State Medical Society, proceedings of, 326
Iron, preparations of, 512
Illinois State Medical Society, transacactions of, 513. 241. 104
Ileo-Colitis, a history of, by S. W. Thompson, M.D. 529
Iodine injections in ovarian dropsy. 455
Infancy and childhood, diseases of, 276
Iodine inhalations in phthisis. 208
Iodine as an antidote to the curare, 183
Iodine as an antidote to the bite of rattlesnake. 187
Institutions for the insane, by Pliny Earle, M D. 323

Letter from Buena Vista Stephenson Co. Ill. 138
La Salle Co. Medical Society. 130
Letter from Paris, 145
Letter from Louisiana, 181

Miscellaneous, 520
Medical education, voluntary system, 525
Morbus coxalgia. 433
Milk-sickness an essay on, by S. W. Thompson, M.D. 385
Molities ossium, preceded by degeneraion of the muscles. 415
Medical Societies. 45. 161. 285, 430
Medical Association of Southern-Central New York. 366
Do do at Aurora. 190
Mercy Hospital Report for 1853. 140
Materia Medica, Parcira's. 141
Medical obituaries. 142
Medical proceedings. 144
Miasmatic diseases, retrospect of, by H. Nance, M.D. 54
Medical Jurisprudence. 43

Notes of Travel. 9
North-Western Med. and Surg. Journal, to the Editors of. 48
Nitrate of soda, medical properties of by J. B. Brown, M D. 61
Natural Philosophy, Lardner's. 237
New Orleans. 316

Ophthalmoscope. 445
Os uteri. complete occlusion of, by B. N. Bond, M.D. 403
Onanism in a boy seven years old, by A. Garwood, M D. 59
Obituary. 98
Opium, poisoning by. 22
Opium in fevers and inflammations, by H. R. Payne, M.D. 289

Psychotherapeia, by W. C. Dendy Esq. 313
Precocious development of sexual system. 15
Probangs for the larynx of silk instead of sponge. 16
Psychical disturbances, treatment in the first stage. 212
Phosphate of lime to promote alimentatation. 216
Pathology, Surgical Lectures. 369
Physical training, public address on, 376
Pneumonia. 277
Personal. 279
Practical Items or chapters on small matters. 436
Potassa fusa and potassa cum calce. 454
Registration of births, deaths &c. 520
Rheumatism and scarlatina. 205
Rush Medical College 136
Report of Joint Committee of Phil. Co. Medical Society. 91. 23
Rectum, prolapsus of. 315

Snake bites, by W. Hanley. M.D. 497
Suture tendons 570
Spina bifida. 556
Skeleton and teeth, forms of. 467
Surgery, the Science and Art of. 417. 47
Stark Co. Med. Society 87
Tracheotomy in epilepsy 549
Turpentine, uses of, by Thomas Hall, M D. 193. 97. 40. 1
Tinct ferri muriatis in scarlet fever. 221
Therapeutics. 175
Tumor obscure and sudden death. 191
Therapeutics and Materia Medica. 47

Vaccine virus. 383
Venereal disease, treatise on. 25

Wound, gun-shot, by Ira E. Oatman, M.D. 301
Wounds, poisoned, by Daniel Brainard, M.D. 337

Yellow fever. 177. 165. 377

### CONTENTS

ORIGINAL COMMUNICATIONS :— PAGE

ART. I.—A History of Ileo Colitis. By S. W. Thompson, M.D. . 529

ART. II.—An Address, read before the Madison County Medical Society November, 1854. By John S. Dewey, M.D., . . . . . . 541

SELECTIONS :

On Tracheotomy in Epilepsy Laryngea, . . . . . . 549

Spina Bifida, . . . . . . . . . . . . . 556

Local Anæsthesia, . . . . . . . . . . 557

Suture Tendons, . . . . . . . . . . . 560

BOOK NOTICES :

Bernard and Robin on the Blood, . . . . . . . . 561

A Treatise on Diaphragmatic Hernia, . . . . . . . . 565

MEDICAL NEWS :

Æsculapian Medical Society, . . . . . . . . 567

EDITORIAL :

Clinical Instruction, . . . . . . . . . . 570

### RECEIPTS DURING OCTOBER, NOVEMBER, & DECEMBER.

### ILLINOIS.

| | | | |
|---|---|---|---|
| Dr W Dodge | 6 00 | Dr M A Cooper | 2 50 |
| B Woodward | 1 00 | O S Johnson | 2 00 |
| S Thompson | 4 00 | L Wallace | 2 00 |
| C L Webster | 6 00 | B Harris | 2 00 |
| J M Logan | 6 00 | J P Russell | 2 00 |
| C H Harrison | 3 00 | J Shreve | 6 00 |
| W J Wackerle | 6 00 | R Sanders | 1 00 |
| A Hull | 2 00 | J R Harrod | 2 00 |
| T C Patterson | 2 00 | M Greenman | 2 00 |
| Norton | 2 00 | S G Mans | 4 00 |
| Naramore | 4 00 | | |

### INDIANA.

| | | | |
|---|---|---|---|
| Drs H M and G Sexton | 6 00 | Dr S Ross | 2 00 |
| J W Green | 2 00 | Holderman | 1 00 |

### IOWA.

| | | | |
|---|---|---|---|
| Drs Barton and Smith | 6 00 | Dr E Hollingsworth | 2 00 |
| W G Elder | 2 00 | S B Harriman | 2 00 |

### MICHIGAN.

| | | | |
|---|---|---|---|
| Dr Stoddard | 2 00 | Dr O P Strowbridge | 6 00 |
| S W Joyce | 4 00 | | |

### WISCONSIN.

| | | | |
|---|---|---|---|
| Dr J H Turner | 1 00 | Dr J H Babcock | 4 00 |
| Rich. Jones | 6 00 | H Russell | 2 00 |
| C Miller | 6 00 | J L Ingersall | 2 00 |

| | |
|---|---|
| Dr A A Hemenway, Oregon Ter. | 2 00 |
| A E Ames, Min. Ter. | 2 00 |

